# Secrets and Patents versus Professional Progress

**Published:** 1898-12

**Authors:** J. Morgan Howe

**Affiliations:** New York.


ARTICLE III.

### SECRETS AND PATENTS VERSUS PROFESSIONAL PROGRESS.

J. MORGAN HOWE, M. D. S., M. D., NEW YORK.

Abstract of a paper read before the New York Institute of Stomatology.

Statements and discussions relating to secrets and to patents have been frequent enough during recent years and months to justify the conclusion that further agitation is timely, and will hasten the establishment of true moral standards.

Whether we call ourselves a profession or claim the connection of a specialty in a kindred profession, our claim to professional character, in any liberal sense, depends not on the nature or kind of work which we do as individuals, but on our relation to and treatment of each other and of the public. Quacks and charlatans work with more or less skill in the same sphere as do honorable members of the professions, but that does not give them professional standing. We began to be a professional body when less than sixty years ago our honorable predecessors organized the first dental society, issued the first dental periodical, and established the first dental college. These institutions were founded to oppose the continuance of isolation among practitioners, to break down the barrier of secrecy that prevailed, and to begin the interchange and general dissemination of knowledge. It was a distinctly altruistic movement, made, despite much opposition, by the most intelligent and prosperous of our craft, who could well have afforded to allow things to remain as they were; for the benefits to accrue were more for the uninstructed and for the coming practitioners than for themselves.

There are men now living and practicing who were in practice at and before that time, and I cannot do better than quote from the recently published "personal recollections" of one of them. Dr. Charles Merritt says of the absence of professional feeling among dentists, "Every new discovery or improvement made was kept a profound secret. No dentist with any reputation would allow another to see him operate, or let him get a peep into his laboratory, fearing that some of his cherished secrets would be stolen. * * * There was no organization of dentists, no real professional history. Each man was a law unto himself and pursued his own independent course, devising his own methods and practicing according to his own knowledge, with no ethical code to restrain him." From such a condition of isolation as this we began to emerge when the instrumentalities were set at work to accumulate a common fund of knowledge, and to establish mutual fraternal relations, obligations and privileges, which would bind us together as a professional body.

We are accustomed to auto-congratulations in regard to the phenomenal progress that dentistry has made during recent years, but we have not always remembered the forces from which these constant advances have received continual impetus. Real professional progress in any line depends upon the general diffusion of a scientific spirit, which promotes to the imparting of information for the benefit of all, no matter how the knowledge is obtained. From knowledge thus received, added to, and in turn imparted, the successive advances are made that raise the level of intelligence of the whole professional body. By such methods the least enlightened has the means of advancement offered him as freely as any of his confreres. Our professional progress has been due to such liberal influences, which have prevailed, notwithstanding a constant tendency on the part of some to attribute that progress to the materials incidental to increased intelligence, instead of to educational advancement coupled with professional sentiment. A recent circular of the New York Academy of Sciences says of the investigations of scientific men, "Their results are freely given to the world for the benefit of all," and it is this characteristic and others of similar nature that must be a dis inguishing mark between the professional man and the man of business who uses his knowledge in manufacture and trade.

All organizations of men necessarily require the individual to relinquish something in return for advantages and benefits received, and to accrue through like action on the part of others. If we consider even so commonplace a relation as that of citizenship, we remember that one is required to give up time to serve the State as jurors and as militamen; to pay money in taxes; and even in some cases to give up our right to do as we wish with our material possessions. In thickly settled communities we are restricted as to how we shall locate our house on our own property, of what material we shall build it, and what the character of the conveniences shall be that we put inside it. All such and many other instances of yielding up of rights and privileges, for which the barbarian would fight, are peacefully conceded for the general good, as civilization advances. And similarly, as the processes of evolution proceed and society becomes more complex, other organized bodies must require concessions peculiar to themselves, which also imply the giving up of rights or possessions in return for benefits conferred. There is no doubt about the legal right of an individual to withhold information regarding any knowledge he may acquire; but if he belongs to a professional body, and the information he retains for his own use relates to the work of his confreres, he is evidently returning to the practice of those who thus withheld knowledge before the organization of the professional body began. Relevant to the provision of the medical code against the use of nostrums, an editorial in the "Dental Cosmos," not much over two years ago objected in the strongest terms to the advertising of nostrums in medical and dental journals, as well as the use of such compounds by practitioners, but all the force of the article was directed against medicinal preparations. Nostrum is the Latin word for "our, ours," and is supposed to be applied, as it is, from "the habit of quacks and other advertisers of claiming special virtue for their wares as 'our own make,' " and, although more commonly applied to medicines, is equally applicable to any scheme or device of a quack or charlatan." I submit that no use of the word could be more appropriate than its application to the secret formulæ of alloys and cements that are offered us in all dental journals. Dr. Ottolengui has also given a valuable contribution on "secret remedies," but he also confined his attention to drug compounds, and ignored all consideration of the compound remedies for dental caries that we are all using constantly. Whether it has occurred to either of these gentlemen, who edit journals for supply houses, that the secret and nostrum character of the alloys and cements, in which their firms and most dealers are interested, is the same as that to which they object in drug compounds, does not appear; but I am sure the logic of their convincing arguments against secret remedies and nostrums will lead most of us to include these filling compounds as differing not at all in principle from other secret preparations.

During the last three years we have had very full and valuable publications from Dr. Black, as the result of his scientific study of amalgams, but as yet it seems as if we were receiving no benefit from the information he has imparted, as every manufacturer and dealer in supplies has his own compound, which he is most anxious to sell.

Dr. Crouse, for the Dental Protective Supply Company, while acknowledging his indebtedness to Dr. Black's publications and suggestions, by adding to these his own investigations, has "solved the problem at last" and produced "the only perfect alloy," but he objects to revealing the results of his studies or the composition of his product. I question the propriety of calling this compound "Fellowship" alloy, because it embodies such a flagrant violation of that professional sentiment which expects members of the body to contribute of their knowledge to the common fund as freely as they have received. Dr. Crouse, in reply to a letter written him in January last, calls the proposition to publish the formulæ of alloys "absurd," and thinks that such publication has been "detrimental to the dental profession," and that it has been a reason for the production of "worthless preparations" and of constant variations in quality. He thinks he can "best help my (his) profession" "not by giving them a formulæ," especially in view of the cost to the Supply Company of a metallurgist, months of time, and expensive instruments. He says, "Why should the conclusions arrived at be made public?" "This course would be manifestly unjust to the stockholders."

The statement is from the manufacturers' standpoint. His interest in maintaining secrecy is frankly admitted, while, on the other hand, he claims it is good for the "profession" to be kept in ignorance; because the "worthless preparations" on the market are the result of the publication of formulæ.

It does not seem as if this reasoning is convincing or acceptable to dentists who regard their vocation as a professional one. We are all very thankful, I am sure, to Dr. Crouse for his services in connection with the Protective Association, but we may decline, I hope without offense, the proffer of a guardianship that would shield us from too much knowledge. On the contrary, it is time in this stage of our maturity to demand such knowledge, even if stockholders have to accept less dividends.

Dr. Flagg and a very few other manufacturers of alloys have published their formula. Dr. Keller published not only the composition of alloys that he made, but also the results of analyses of sixty-five secret compounds then upon the market. There would seem to be no objection possible to the preparation and sale of known compounds by a professional man to his confreres. Painstaking care and skill in such work —as in everything—is worth paying for; but such a question by a dentist as "Why should the conclusions arrived at be made public ?" takes us back to early days. It is the same question in effect that those men asked who opposed the efforts of Hayden, Harris, Parmly, Flagg and others who began the the agitation for the establishment of our professional relations. It has a tone of antiquity about it that in these days of societies and journals sounds strangely coming from a member of a profession. Although paradoxical, it shows that we are a youthful body. Our willingness to continue buying and using secret compounds, for which extravagant claims are made, must be due to mental qualities similar to those which keep up the sales of quack medicines to the general public. But those who claim to be professional men should certainly rise above such a level. We cannot blame the proprietors for taking advantage, as business men, of the desire to buy what they have for sale; but to argue that it is not good for professional men to know what they are using is certainly unique. Of course, it is recognized as an easy matter to analyze any inorganic compound, so that the composition may certainly be known, if it is really desired; but the question of interest just now is, What relation does a so-called professional man hold to his fellows who withholds information for gain ? I contend there is as much justification for expecting and demanding the giving up of secrets as there is that members of a professional vocation should conform in any other way to the concensus of opinion concerning what is favorable to progress. There is no more right or reason in an interdiction against public advertising than against making, selling, or holding an interest in a secret nostrum, for both are an injury to the profession as such. The moral status of any course of action may be justly estimated by calculating the effects of such conduct on the whole body, if all its members resorted to similar behavior. Applying such a test to secrecy, the conclusion must be, if it would be an injury to the professional body for all our members to withhold information, to reserve for their personal benefit everything they can turn to their own advantage, and cease all efforts to enlighten for the sake of elevating professional status; then there is no question that he who claims exemption from the liberal usage—which brought us out of the chaotic condition of former days—is working an injury to the extent of his influence.

Solicitude has been recently expressed in regard to the increase and success of the so-called "dental parlors," conducted with the brazen advertising of "painless operation and flaunting college degrees. Why should these men be asked to give up their legal right to use such means as they find of pecuniary advantage? They have as good reason for claiming their right to do as they please in their method of obtaining business as have those who in any other way seek their own interests, without regard to their effects upon the profession of which they claim to be members. Their methods are forbidden because they injure the professional body. The right must be relinquished for the general good. A general desire among dentists to know the composition of all the compounds they use, and a determination to deliver themselves from their present bondage, together with the establishment of an antisecret ethical standard by dental societies and schools, will do more than anything else to make publications, such as Dr. Black's, of service to all dentists, and at the same time make all dentists more intelligent and better professional men. It is an entirely practical proposition that we should have compound filling-materials made from known formula by experts, who will serve us as willing as pharmacists now serve physicians; and the practical way to bring about the change is for dentists to cease using secret compounds. Publication of formula will be general when it is found to pay. Chemists and metallurgists will fill orders when they are employed.

